# Molecular characterization of Human T-cell Lymphotropic Viruses 1 and 2 (HTLV-1 and HTLV-2) in samples from blood donation candidates in the state of Pará, Brazilian Amazon

**DOI:** 10.1016/j.bjid.2025.104577

**Published:** 2025-09-10

**Authors:** Carolina Alcântara Maneschy, Patricia Santos Lobo, Carlos Eduardo de Melo Amaral, Katarine Antonia dos Santos Barile, Jairo Augusto Americo de Castro, Rodrigo Vellasco Duarte Silvestre, Felipe Bonfim Freitas, Luana Silva Soares, Sylvia Fátima Santos Guerra

**Affiliations:** a Instituto Evandro Chagas, Sessão de Virologia, Belém, PA, Brazil; b Centro de Hemoterapia e Hematologia do Pará, Gerência de Biologia Molecular, Belém, PA, Brazil

**Keywords:** HTLV-I infections, HTLV-II infections, Phylogenetic analysis, Molecular epidemiology

## Abstract

The Human T-Lymphotropic Virus (HTLV) is a retrovirus belonging to the Retroviridae family and the Deltaretrovirus genus, which has a tropism for T lymphocytes. Despite being the first human retrovirus identified, knowledge about its infection remains limited, highlighting the need for further research, especially in Northern Brazil.. This study aimed to phylogenetically characterize HTLV-1 and HTLV-2 samples identified in blood donors from the state of Pará. The phylogenetic characterization was performed using DNA samples from blood donors at the HEMOPA Foundation, collected between January 2015 and December 2021. The 5′LTR regions of HTLV-1 and HTLV-2 were amplified using specific primers with Nested PCR. The amplified products were purified and sequenced using the dideoxynucleotide method, with the same primers used in the Nested PCR, for subsequent phylogenetic analysis and the construction of a sequence database, as well as deposits in GenBank. The phylogenetic analysis of the 5′LTR sequences of HTLV-1 from this study, compared with isolates available in GenBank, showed that 100 samples clustered with Cosmopolitan subtype isolates, Transcontinental subgroup, while 2 samples clustered with Cosmopolitan subtype isolates, Japanese subgroup. Regarding the phylogenetic analysis of HTLV-2 from this study in comparison with other GenBank isolates, most of the isolates clustered with strains described as HTLV-2c, and only three samples clustered with strains described as HTLV-2a. The epidemiological profile found in the analyzed samples consisted mainly of women, with an average age of 40 years and a low level of education. The molecular characterization of the viral genome provides information about the viral subtypes circulating in the population. This information may be important for increasing investments in screening and monitoring individuals infected with HTLV, thereby contributing to prophylactic practices related to infection and the dissemination of knowledge about HTLV.

## Introduction

Human T-Lymphotropic Virus Types 1 and 2 (HTLV-1 and −2) belong to the *Retroviridae* family, *Orthoretrovirinae* subfamily, and *Deltaretrovirus* genus^1^ HTLV is an enveloped virus with an icosahedral nucleocapsid. Its genome consists of two single strands of positive-sense RNA and contains the *gag, pol*, and *env* genes, a sequence near the 3′ end known as the *pX* region, and Long Terminal Repeats (LTRs) at both the 5′ and 3′ ends of the genome^2^, ^3^, ^4^ HTLV-1 and HTLV-2 share approximately 70 % nucleotide similarity and have a similar genome structure, with differences primarily observed in the *pX* gene.^2^^,^^3^

HTLV-1 infection is associated with serious diseases such as Tropical Spastic Paraparesis/HTLV-1 Associated Myelopathy (TSP/HAM) and Adult T-cell Leukemia/Lymphoma (ATLL), as well as conditions like infectious dermatitis, Sjögren's syndrome, and uveitis^3^^,^^5^^,^^6^ Although HTLV-2 is less associated with diseases, some studies link the infection to neurological manifestations and predisposition to bacterial infections^3^^,^^7^, ^8^, ^9^, ^10^

So far, seven subtypes of HTLV-1 have been described, namely HTLV-1a to HTLV-1 g^11^, ^12^, ^13^, ^14^, ^15^, ^16^, ^17^, ^18^ Most infections are caused by subtype a, referred as Cosmopolitan^19^ It is estimated that the approximate number of people infected with HTLV-1 worldwide is between 5 and 10 million, with the highest prevalence in Japan, the Caribbean, South America, sub-Saharan Africa, certain areas in the Middle East, and Australasia^20^ In Brazil, the number of people infected with HTLV-1 is approximately 2.5 million, with lower prevalence in the South region and higher prevalence in the North and Northeast regions^21^, ^22^, ^23^

HTLV-2 is currently classified into four subtypes, named HTLV-2a to HTLV-2d The estimated number of people infected with HTLV-2 worldwide ranges from 670,000 to 890,000,^24^ and it is commonly found in indigenous populations in Central and South America, as well as in studies involving intravenous drug users^25^, ^26^, ^27^, ^28^, ^29^, ^30^

In the state of Pará, several epidemiological studies were carried out on the prevalence of antibodies against HTLV-1/2 in a variety of specific groups. Study carried out by GUERRA et al. (2018),^31^ with pregnant women in the city of Belém, detected a seroprevalence of HTLV-1/2 of 0.6 %. Another study, carried out by MANESCHY et al. (2022)[22] in a population of blood donors described a seroprevalence of HTLV of 0.2 %, this study also demonstrated the highest prevalence, among blood donors, in the North and Northeast regions when compared to other Brazilian regions. Lospes et al. (2022)^32^ carried out a study with individuals from different neighborhoods of Belém, capital of the state of Pará, demonstrating an HTLV seroprevalence of 0.3 % in this population.

The risk of HTLV transmission through blood transfusions has prompted various countries to implement screening for this virus among blood donors to ensure transfusion safety. Accordingly, the Ministry of Health, in its ordinance n° 1376, dated November 19, 1993, made it mandatory to test for anti-HTLV-1/2 antibodies in blood banks in Brazil.^33^

Considering the average seroprevalence of the infection among blood donors in the state and the efficient transmission of HTLV through blood transfusions, the importance of developing research focused on the molecular characterization of this retrovirus among the blood donor population is reaffirmed, aiming to contribute to the knowledge regarding the presence and characterization of HTLV in the Northern region of Brazil.

## Material and methods

### Ethics approval

The present study was submitted to Plataforma Brazil, the national and unified database for registering research involving human subjects within the CEP/CONEP system. A waiver of the Informed Consent Form (ICF) was requested, due to the absence of identification and/or disclosure of confidential information, as only secondary data already available in blood bank systems were used. These data were previously authorized by the donor for the required tests at the time of donation. The study was approved under opinion number: 5.639.621.

### Sample collection and molecular characterization

All blood donor samples from the Pará State Center for Hematology and Hemotherapy (HEMOPA), were analyzed, including individuals of both sexes, aged over 18 years, who showed reactivity to HTLV in the serological screening stage and were molecularly confirmed for HTLV-1 or HTLV-2 infection, between January 2015 and December 2021, totaling 109 samples.

The proviral DNA used in the analysis was previously extracted from the 109 total blood samples of anti-HTLV-1/2 antibodies seropositive donors using the PureLink™ Genomic DNA Kit (Invitrogen ‒ Life Technologies, Carlsbad, CA, USA), following the manufacturer's instructions. It was then amplified for the non-homologous regions of the *pol* gene of HTLV-1 and HTLV-2 using an in-house qPCR protocol. The samples were stored at −20 °C, properly identified, and processed in the Virology section of the Evandro Chagas Institute.

### Molecular characterization of the positive samples

The 5′LTR regions of HTLV-1 and HTLV-2 were amplified with specific primers using a Nested PCR as described in a protocol standardized by Vallinoto et al. 200,2^34^ The reactions for amplifying the 5′LTR region were conducted in a final volume of 25 µL. In each amplification reaction, after an initial denaturation at 95 °C for 5 minutes, 35 cycles of 30 seconds at 95 °C, 30 seconds at 62 °C, and 40 seconds at 72 °C were performed. The 35 cycles were followed by a final extension of 10 minutes at 72 °C. In the subsequent amplification step (Nested PCR), 2.0 µL of the product from the first amplification was used under the same reaction conditions.

The products of the Nested PCR amplification were analyzed by electrophoresis on a 1.5 % Agarose Gel in TBE buffer pH 8.4 (89 mM Tris; 89 mM boric acid; 2 mM). The run was conducted in an electric field of 110 Volts (V) for 30 minutes, followed by visualization under UV light and image capture using an image capture system (Vilber Lowmart). The products showing amplification of the 5′LTR region (744 for HTLV-1 and 788 for HTLV-2 bp) were purified and subjected to sequencing reactions for further nucleotide base analysis and phylogenetic tree construction.

### Sequencing

The amplified products were purified and sequenced using the same primers used for the Nested PCR along with the BigDye Terminator v.3.1 Cycle Sequencing Kit (Applied Biosystems, CA, USA). The samples were sequenced for phylogenetic analysis and to build a sequence database, as well as to deposit them in GenBank (https://www.ncbi.nlm.nih.gov/genbank).

Sequencing was performed using the dideoxynucleotide method on an ABI Prism 3130 × *l* Automatic Sequencer (Applied Biosystems). The products were subjected to a temperature of 96 °C for 1 minute once, followed by cycling at 96 °C for 15 seconds, 50 °C for 15 seconds, and 60 °C for 3 minutes, repeated 30 times. Subsequently, this sequencing reaction was purified by ethanol/isopropanol precipitation and resuspended in formamide.^35^

### Analysis of nucleotide sequences

The partial sequences of the 5′LTR region of HTLV-1 and HTLV-2 obtained were assembled and edited using the Geneious program (v.9), and then aligned using the Mafft algorithm (v.7) with HTLV-1 and HTLV-2 samples deposited in the GenBank database (http://www.ncbi.nlm.nih.gov) included via the BLAST tool. The HTLV-1 samples from this study were compared with the prototype strain HTLV-1_ATK_^36^ (J02029) and the HTLV-2 samples were compared with the prototype strains HTLV-2_MO_^37^ (M10060) of subtype 2a and HTLV-2_NRA_^38^ (L20734) of subtype 2b

For the phylogenetic analysis, the Maximum Likelihood method was adopted using the FastTree software v. 2.1.11, including the GTR + Gamma + F substitution model and bootstrap reliability testing configured for 1000 replicas indicating node support values in the clades. The phylogenetic dendrograms were visualized using the Figtree program.

The nucleotide sequences identified in this study were deposited in GenBank with accession numbers PPP537793-PP537894 for HTLV-1 and PP266323-PP266347 for HTLV-2.

HTLV-1 sequences that showed 100 % similarity to other samples were excluded from the phylogenetic tree to avoid redundancy. Additionally, sequences with near-complete similarity and identical epidemiological profiles were also excluded. As a result, 75 unique HTLV-1 sequences were included. For HTLV-2, all sequenced samples were analyzed.

## Results

**Fig. 1:**
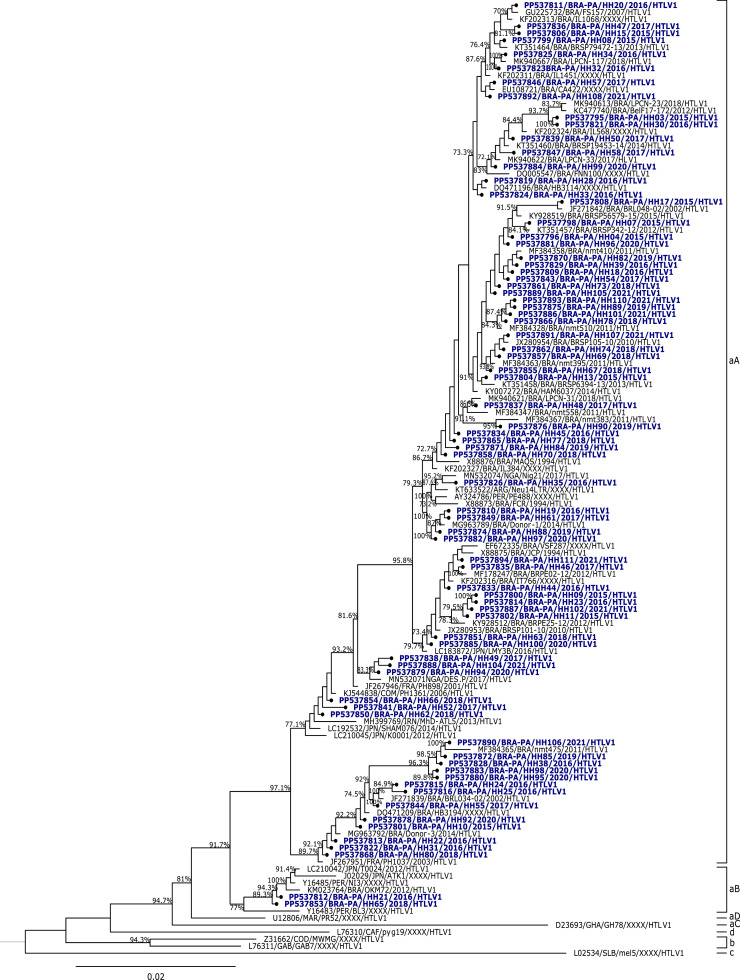
Phylogenetic tree of HTLV-1, based on the nucleotide sequence of the gene encoding the 5′LTR region. Bootstrap values are indicated at the nodes of the tree, with blue referring to samples from this study.

In this study, 84/109 (77 %) samples of HTLV-1 and 25/109 (23 %) samples of HTLV-2 were characterized. The analysis of the nucleotide sequences of HTLV-1 isolated in this study demonstrated an average similarity of 99 %, and when compared to the prototype HTLV-1ATK, it showed a similarity of 97.2 %. The phylogenetic analysis of the 5′LTR sequences of HTLV-1 from this study and some isolates described in the literature showed that 82 samples clustered with isolates of the Cosmopolitan subtype, Transcontinental subgroup (aA), and 2 samples clustered with isolates of the Cosmopolitan subtype, Japanese subgroup (aB), with bootstrap values of 97.1 % and 89.3 %, respectively (Fig. 1).

The samples grouped in the Cosmopolitan subtype, Japanese subgroup came from first-time donation candidates residing in the Metropolitan Region of Belém, who showed reactive results for HTLV-1/2 in their first donation. One sample is from a 49-year-old married woman of mixed race, who completed secondary education and made a voluntary donation. The other sample is from a 30-year-old single male donor of white race, who also completed secondary education and made a linked donation.

**Fig. 2:**
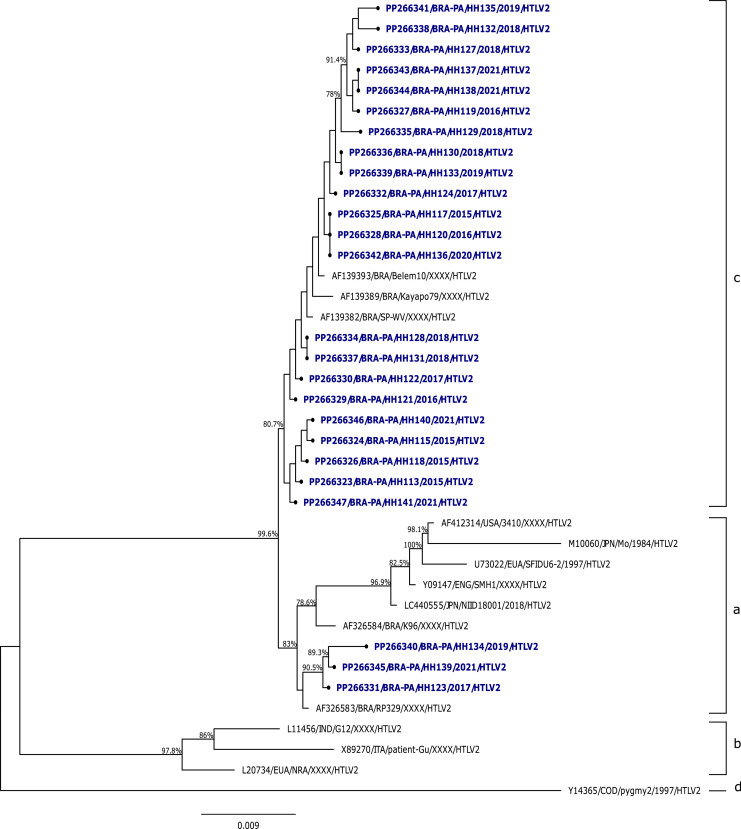
Phylogenetic tree of HTLV-2, based on the nucleotide sequence of the gene encoding the 5′LTR region. Bootstrap values are indicated at the nodes of the tree, with blue referring to samples from this study.

Regarding HTLV-2 samples, the analysis among the sequences isolated in the study showed an average similarity of 99.8 %, and the comparison with prototype strains HTLV-2_MO_ and HTLV-2_NRA_ showed percentages of 97.6 % and 94.7 %, respectively. In relation to the phylogenetic analysis of the 5′LTR region sequences of HTLV-2 from this study with other isolates described in the literature, it was evidenced that most of the isolates (22/25) clustered with strains described as HTLV-2c, with a bootstrap value of 80.7 % and average identity of 99.8 %, 99.7 %, and 99.6 % with the samples Belem10, Kayapó70, and SP-WV, respectively. Only 3 samples clustered with strains described as HTLV-2a, with a bootstrap value of 83 % and average identity of 97.8 % compared to the prototype strain HTLV-2_MO_ (Fig. 2).

The epidemiological profile found among blood donation candidates in Pará was described according to each subtype and subgroup identified in the study. It was noted that the highest frequency was among women from the Metropolitan Region of Belém, of mixed race, with a low level of education and first-time donors, as described in Table 1.

**Table 1:** Epidemiological profile based on viral subtype and subgroup.

Variables	Total	HTLV-1aA		HTLV-1aB		HTLV-2a		HTLV-2c	
		n	%	n	%	n	%	n	%
Sex									
Female	74	58	71	1	50	2	67	13	59
Male	35	24	29	1	50	1	33	9	41
Geographical origin									
Downstream Amazon rive	3	3	4	‒	‒	‒	‒	‒	‒
Marajó	1	1	1	‒	‒	‒	‒	‒	‒
Belém Metropolitan Region	68	45	55	2	100	1	33	20	91
Northeast	10	8	10	‒	‒	‒	‒	2	9
Southeast	26	24	29	‒	‒	2	67	‒	‒
Southwest	1	1	1	‒	‒	‒	‒	‒	‒
Marital Status									
Married	37	27	33	1	50	‒	‒	9	41
Single	63	50	61	1	50	3	100	9	41
Divorced	5	4	5	‒	‒	‒	‒	1	4
Widowed	4	1	1	‒	‒	‒	‒	3	14
Skin color									
White	14	9	11	1	50	‒	‒	4	18
Brown	93	72	88	1	50	3	100	17	77
Black	2	1	1	‒	‒	‒	‒	1	5
Level of Education									
First grade	27	24	29	‒	‒	‒	‒	3	14
Second grade	69	47	57	2	100	2	67	18	82
Third grade	10	8	10	‒	‒	1	33	1	4
Postgraduate	3	3	4	‒	‒	‒	‒	‒	‒
Type of donor									
Sporadic	16	11	13	‒	‒	2	67	3	14
First time	86	67	82	2	100	1	33	16	72
Return	7	4	5	‒	‒	‒	‒	3	14
Type of donation									
Spontaneous	75	56	68	1	50	2	67	16	73
Linked	34	26	32	1	50	1	33	6	27

Regarding the age of the group classified in the Transcontinental subgroup (HTLV-1aA), an average of 40 years was observed, with a minimum of 18 years and a maximum of 65 years. For the Japanese subgroup, the average was also 40 years, with minimum and maximum ages of 30 and 49 years, respectively. Concerning the subtypes of HTLV-2, the samples classified as HTLV-2c showed an average age of 44 years, with a minimum of 19 years and a maximum of 64 years. The samples described as HTLV-2a had an average of 38 years, with a minimum age of 26 years and a maximum of 51 years.

## Discussion

Through the molecular analysis of the 5′LTR region of HTLV, it was possible to define the circulating HTLV subtypes among the blood donation candidates at the HEMOPA Foundation during the study period.

Most HTLV-1 samples were included in the clade of the Cosmopolitan subtype, Transcontinental subgroup (HTLV-1aA) in the phylogenetic tree, alongside samples from Argentina, Peru, Russia, France, Nigeria, Cameroon, Iran, Japan, and other samples from different states in Brazil, such as Amazonas, Pará, Bahia, Ceará, Pernambuco, and São Paulo.

Thus, it was possible to confirm the higher prevalence of the HTLV-1aA genotype, which is consistent with the global distribution pattern of HTLV-1, in which the Cosmopolitan subtype (1a) is the most widely disseminated worldwide, having been identified across several continents, including South America, Europe, Africa, and parts of Asia.^20^^,^^39^, ^40^, ^41^, ^42^ The Cosmopolitan subtype, Transcontinental subgroup, is particularly prevalent in populations with a long history of migration, such as in northern Brazil. This may be partly related to the high frequency of this genotype observed in the metropolitan region of Belém, considering that a similar infection profile has been reported in other comparable urban settings in developing countries, particularly in Latin America.^40^

The molecular characterization of HTLV-1 in blood donation candidates in the State of Pará was previously conducted by Santos et al. (2009),^43^ where all sequenced HTLV-1 samples were classified as belonging to the Cosmopolitan subtype, Transcontinental subgroup (HTLV-1aA). Another study at the Blood Center of the State of Piauí, conducted by Ribeiro et al. in 2018,^29^ involved blood donation candidates between 2011 and 2012, where the authors reported that all HTLV-1 isolates were classified as belonging to the Cosmopolitan subtype (1a), Transcontinental subgroup (A).

In blood donors from Argentina, in the province of Corrientes, HTLV-1 isolates of the Cosmopolitan subtype were exclusively grouped within the Transcontinental subgroup.^41^ A study conducted in Mozambique revealed that all detected HTLV-1 isolates also belonged to the Cosmopolitan/Transcontinental subtype, demonstrating the global reach of this genotype.^42^

In the present study, two samples, one from a male and one from a female, clustered with the Cosmopolitan subtype, Japanese subgroup (HTLV-1aB), previously described by Vallinoto et al. (2004)^44^ in Japanese immigrants residing in Tomé-Açú-PA, in Japanese descendants residing in Campo Grande-MS by Bandeira et al. (2015),^45^ and residents in São Paulo^46^ However, in this study, it was detected in donation candidates from the Metropolitan Region of Belém who did not report a recent travel history or declared self-identified yellow race, factors that could be associated with possible Japanese descent or immigration.

This subgroup is considered endemic in Japan and is rarely identified outside of this ethnic and cultural context. Its presence in a mixed population with no apparent direct links to Japanese immigration suggests possible new routes of viral introduction, likely associated with silent transmission chains. Furthermore, this finding may indicate underreporting of migratory connections or may reflect the increasingly intense population movement between urban regions.

Although it is difficult to specify the transmission route of HTLV-1aB among the donation candidates, this study clearly shows the epidemiological importance of ongoing investigations into the emergence of new infectious agents in new geographic areas, especially considering that Belém likely represents the main entry point for this virus in the Brazilian Amazon.^43^

Among the HTLV-2 samples analyzed in this study, all clustered with other strains described as HTLV-2c. The GenBank isolates classified in subtype 2c, along with the samples from this study, corroborate descriptions in the literature referring to this subtype as exclusive to Brazilian populations and prevalent among Amerindians, as it includes samples from Amerindians of the Kayapó tribe (Kayapo79), blood donors from the State of Pará (Belem10, HH113-HH141), and one co-infected individual (HTLV-2/HIV-1) (SP-WV).^30^, ^46^, ^47^

The study by Santos et al. (2009),^43^ conducted with blood donor populations, first reported a sample of HTLV-2b and 19 samples classified as HTLV-2c in the Brazilian Amazon. Another study involving blood donors conducted phylogenetic analysis of the LTR region of HTLV-2, describing that the 11 isolates analyzed belonged to subtype HTLV-2a/c.^29^

The Brazilian Amazon is the largest endemic area for HTLV-2c, and the occurrence of this subtype in the Brazilian population, especially in the Amazon region, can be attributed to the migratory events that populated the area. Vallinoto and Ishak (2017)^30^ suggest that extensive miscegenation, which occurred during the colonization period and the formation of Amazonian populations, resulted in the introduction of HTLV-2c into emerging urban communities, as a consequence of population expansion.^28^^,^^30^

The epidemiological profile found in the samples analyzed from blood donation candidates in the State of Pará was consistent with previously described literature, showing the highest prevalence among women, with an average age of 40 years and a low level of education, as well as the highest frequency in the Metropolitan Region of Belém, which may be related to the greater number of donations in this region.^20^^,^^48^, ^49^, ^50^

It was not possible to identify a difference between the sociodemographic profiles of each HTLV-1 and HTLV-2 subtypes, which may be explained by the sample sizes for each subtype being too disparate to allow for a specific profile association.

This study presents limitations inherent to the analyzed population profile, as it is restricted to blood donors who, at the time of clinical screening, declared not to engage in behaviors considered risky for Sexually Transmitted Infections (STIs). This characteristic may introduce a selection bias, limiting the generalizability of the findings to other populations. Therefore, the results obtained should be interpreted considering the specific context of the individuals evaluated.

HTLV is a neglected etiological agent in Brazil; despite many advances since its discovery, knowledge about it among healthcare professionals and the Brazilian population in general is still insufficient. Therefore, the importance of studies of this nature is evident, as the molecular characterization of the viral genome, based on sequencing combined with bioinformatics analysis, provides information about the circulating viral subtypes in the population.

This information may be crucial for increased investment in screening and monitoring people infected with HTLV, thereby contributing to raising awareness about HTLV.

## Conflicts of interest

The authors declare no conflicts of interest.
